# Ultrasonic vocalisations in the Flinders Sensitive Line rat, a genetic animal model of depression

**DOI:** 10.1017/neu.2024.61

**Published:** 2025-01-23

**Authors:** Linda Marie Kai, Lia Parada Iglesias, Kadri Kõiv, Jaanus Harro, Gregers Wegener

**Affiliations:** 1 Translational Neuropsychiatry Unit, Department of Clinical Medicine, Aarhus University, Aarhus, Denmark; 2 Institute of Genomics, University of Tartu, Tartu, Estonia; 3 Division of Neuropsychopharmacology, Institute of Chemistry, University of Tartu, Tartu, Estonia; 4 Department of Affective Disorders, Aarhus University Hospital–Psychiatry, Aarhus, Denmark

**Keywords:** Rats, anhedonia, vocalisation, depression, reward

## Abstract

**Objective:**

Ultrasonic vocalisations (USVs) emitted by rats may reflect affective states. Specifically, 50 kHz calls emitted during juvenile playing are associated with positive affect. Given that depression is characterised by profound alterations in this domain, we proposed that USV calls may configure a suitable tool for assessing depressive-like states. Utilising the Flinders Sensitive Line (FSL), a well-established animal model of depression, we assessed USV calls emitted by rats during tickling, a procedure based on juvenile rats’ rough-and-tumble play.

**Methods:**

Juvenile FSL rats and their control counterparts, the Flinders Resistant Line (FRL) and Sprague Dawley, were submitted to tickling sessions to imitate rats playing behaviour. The rats were tickled daily for 6 weeks starting at PND21. Tickling sessions were recorded for further acoustic analysis of 50 kHz calls.

**Results:**

Tickling increased 50 kHz calls in all the strains. FSL rats emitted more calls than control strains and exhibited a higher number of flat-trill combination calls.

**Conclusion:**

Tickling is a robust method for inducing 50 kHz USV calls. Analysing USV calls emitted during tickling configurates a suitable method for studying affective states relevant to depression. FSL rats did not present anhedonia but rather higher reward sensitivity, which may underlie their stress vulnerability.

Significant outcomes

- Tickling induced 50 kHz USV calls in FSL and SD young rats.
- Tickling induced a higher 50 kHz USV response in FSL than SD or FRL.
- USV pattern can be used to infer emotional states in rodents.
- FSL does not seem to be a suitable model for studying anhedonia.
- FSL may serve as a model of non-adaptive reward sensitivity.

Limitations

- Animals were single-housed to increase playing behaviour during tickling sessions.

## Introduction

Depression is a severe and life-threatening disease with extensive personal and societal costs (Whiteford *et al*., Whiteford *et al*., 2013). Despite the recent advancements (Krystal *et al*., 2024), current pharmacological treatments are unsatisfactory as most present a delayed onset, limited efficacy and poor long-term symptom control (Dwyer & Duman, 2013, Gigliucci *et al*., 2013). Thus, there is an urgent need to refine the preclinical methods used to discover putative antidepressants (Cryan & Lucki, 2002, Neumann *et al*., 2011, Gururajan *et al*., 2019). The forced swim test is a predictive tool to assess antidepressant-like behaviour (Porsolt *et al*., 1977a; Porsolt *et al*., 1977b; Porsolt *et al*., 1978, Cryan & Lucki, 2002, Cryan & Slattery, 2007, Slattery *et al*., Slattery & Cryan, 2012). Despite being highly popular due to its feasibility and reproducibility, its use, interpretation and conceptualisation are criticised.

Rats emit ultrasonic vocalisations (USVs), inaudible to the human ear. These reflect the affective state of the rats (Knutson *et al*., 1999). For instance, when rats have their tails pinched or are exposed to other aversive stimuli, they elicit 22 kHz vocalisations associated with negative affect (Panksepp, 1999, Knutson *et al*., 2002). Similarly, maternal separation induces calls of 40 or 60 kHz (Boulanger-Bertolus *et al*., 2017; Kaidbey *et al*., 2019). Alternatively, administration of amphetamine either systemically or directly in the nucleus accumbens, a vital structure of the reward system, was linked to 50 kHz vocalisations (Burgdorf *et al.,* 2001; Burgdorf & Panksepp, 2001). These vocalisations are also spontaneously elicited when juvenile rats play with each other, suggesting its association with positive affect (Panksepp, 1999, Knutson *et al*., 2002). The experimenter can imitate the juvenile rats’ rough-and-tumble play through a tickling-like stimulation, increasing 50 kHz vocalisations. The number of calls elicited can be used to infer depressive and anxiety-like behaviour (Burgdorf *et al.,* 2001; Mallo *et al.,* 2007).

The Flinders Sensitive Line (FSL) is an inbred line widely validated as a model of depression (Overstreet, 1992, Overstreet *et al*., 2013). Studies on face validity demonstrate: (I) reduced sucrose intake following stress and increased immobility in the forced swim test, suggesting a depressive-like phenotype (Wegener G., 2012); (II) elevated REM sleep and impairments in the novel object recognition task, modelling disturbed sleep and cognitive deficits related to depression (Wegener *et al.,* 2012). Studies on construct validity in this model show serotonergic, cholinergic, neurotrophic and morphological abnormalities, which have been implicated in depression in humans (Overstreet & Wegener, 2013; Strenn *et al*., 2015; Ardalan *et al*., 2017; Kirkedal *et al*., 2019; Tillmann & Wegener, 2019, Treccani *et al*., 2019; Abildgaard *et al*., 2021; Arjmand *et al*., 2023; Domingos *et al*., 2024). Emphasising its predictive validity, the FSL rat shows decreased immobility in the forced swim test when treated chronically and acutely with drugs that have antidepressant effects in humans (du Jardin *et al*., 2016; Marchetti *et al*., 2020). Therefore, the present study examined the emission of USVs during tickling stimulation in the FSL rat compared to the Flinders Resistant Line (FRL) and outbred Sprague Dawley (SD) rats – both commonly used controls for the FSL rat.

The aims of the study were to i) characterise and ii) investigate the applicability of USVs as a screening method for depressive-like behaviour in a rodent model of depression. It was hypothesised that the depressive-like phenotype of the FSL rat would be reflected in the ultrasonic response to tickling stimulation.

## Methods

### Animals

Juvenile 22-day-old male FSL (n = 27), FRL (n = 24) and SD (n = 23) rats were obtained from the in-house breeding colony at the Translational Neuropsychiatry Unit, Aarhus University (Denmark). Animals were housed at 22 ± 2^o^C and kept at a 12-hour light/dark cycle (lights on at 07:00) with access to food and water *ad libitum*. The rats had access to a hide, nesting material and a wooden stick throughout the experiment. All animal procedures were approved by the Danish National Committee for Ethics in Animal Experimentation (permission ID: 2012-15-2934-00254). Baseline characteristics of the animals are given in Table 1.

**Table 1.** Group characteristics. Group distributions of age and weight at the beginning of the experiment. Values are expressed as means ± SD. FSL = Flinders Sensitive Line rat, FRL = Flinders Resistant Line rat, SD = Sprague Dawley

	FSL		FRL		SD	
Group	Tickled	Light-touch	Tickled	Light-touch	Tickled	Light-touch
Group size	16	8	16	11	16	7
Age (days)	21.6 (±1.1)	21.9 (±1.4)	21.5 (±0.8)	21.9 (±0.8)	23 (±0.8)	22.8 (±0.9)
Weight (day 3)	54.6 (±7)	53.2 (±5.6)	61.8 (±9)	56.6 (±13)	70.3 (±18)	72 (±19)

### Experimental design

On postnatal day 21, the rats were weaned from their mothers. All rats were single-housed during the entire experimental period to increase play behaviour during tickling sessions (Panksepp, 1981; Panksepp *et al*., 1984). The rats were randomly assigned to the experimental groups (tickling/light-touch). All experimental procedures were carried out in the rat’s light cycle.

### Tickling

The rats were tickled daily for 6 weeks starting from postnatal day 21. A tickling session consisted of 15 s acclimatisation to the tickling cage followed by 15 s hand-play with the experimenter, touching the nape of the neck and abdomen, then 15 s of no stimulation followed by 15 s tickling. During the last three seconds of each bout of tickling, attempts were made to pin down the rat and vigorously tickle the abdomen. This method has been described elsewhere (Knutson *et al*., 1998; Mallo *et al*., 2007). A dorsal light-touch group was used as a control group for the tickled rats. As previously described, light touch has been used as a control for tickling, as it is a discernible stimulation but presumably with less reward value (Burgdorf *et al.,* 2001; Burgdorf & Panksepp, 2001; Yamamuro *et al.,* 2013). Acoustic foam was used to isolate the Plexiglas container where the tickling took place (Brudzynski & Pniak, 2002). The same experimenter conducted all the tickling sessions and was blinded to the strain of the rats.

#### Acoustic data acquisition, analysis and classification

The tickling sessions were recorded using Avisoft-RECORDER USGH (v.4.2 Avisoft Bioacoustics, Berlin, Germany). A condenser microphone CM16/CMPA (from AvisoftBioacoustics) was secured 20 cm from the cage floor, and its signal was fed to the Avisoft UltraSoundGate 416H with a sampling rate of 750 kHz. Acoustical analysis was performed with the Avisoft SASlab Pro (v.5.2 Avisoft bioacoustics, Berlin, Germany), and spectograms were generated with a fast Fourier transformation (FFT)-length of 256 points and an overlap of 75% (FlatTop window, 100% frame size). A semi-automatic recording of call parameters was used for the quantitative analysis of the 2-minute tickling or light-touch sessions. In accordance with Reno *et al*. (2013), calls with a frequency between 30–90 kHz were considered 50 kHz calls and between 20 and 30 kHz were considered 22 kHz calls (Reno *et al*., 2013). Furthermore, calls were regarded as individual vocalisations when separated by at least 20 ms. The minimum call length was set at 5 ms and sounds shorter than this were considered noise. For the 50 kHz calls, the differences in morphology and call subtypes were based on the classification by Wright et al. (Wright *et al*., 2010). This classification consists of 14 call categories differently modulate by diverse stimuli, environmental conditions and inter-individual differences, ultimately allowing a more refined analysis of the affective state (Wright *et al*., 2010). The qualitative evaluation of the calls was carried out on the first 15 s stimulations of experimental day 23, as this was the day with the largest quantity of calls. The categorisation was made manually based on the predefined frequency patterns described by Wright et al.

### Data analysis and statistics

All statistics were conducted using SPSS version 22 and GraphPad Prism 10 for Windows (GraphPad Software, San Diego, CA). The statistical analysis of differences between periods of tickling versus periods without tickling and among ticking sessions was done with repeated-measures one-way analysis of variance (ANOVA) with planned post hoc pairwise comparisons using Geisser-Greenhouse correction for sphericity and Bonferroni for multiple comparisons. The statistical analysis of differences between the tickling and the light-touch groups or strain differences for call categories was done using a two-way ANOVA with post hoc Bonferroni for multiple comparisons. Spearman’s correlation coefficient was used to determine the inter-individual stability of calls between days. The qualitative differences in calls between strains were analysed by a one-way ANOVA, followed by a Tukey post hoc test. A two-tailed p-value of less than 5% was considered significant for all tests.

## Results

### Ultrasonic vocalisations

More than 71.000 calls were counted, and more than 2.100 were classified according to Wright et al., categories (Wright *et al*., 2010). As 22 kHz calls accounted for less than 2.9 % of calls on day 1 and 0.6 % on day 6, these were not further analysed. Also, 40 of the 74 rats produced calls which could not be classified as 50 kHz as their frequency was below 30 kHz. They did not have the appearance of 22 kHz either, as they were shorter (<300 ms) or presented frequency modulations. This type of call may be reminiscent of infants’ vocalisations. They were primarily present in the first days of recording, after which they decreased. On experimental day 13, only three rats produced these calls, and they were not further analysed. Finally, in the qualitative evaluation of the call profiles, 26% of the calls could not be classified and went into a miscellaneous group.

### Tickling increases the production of 50 kHz calls

**Figure 1.**
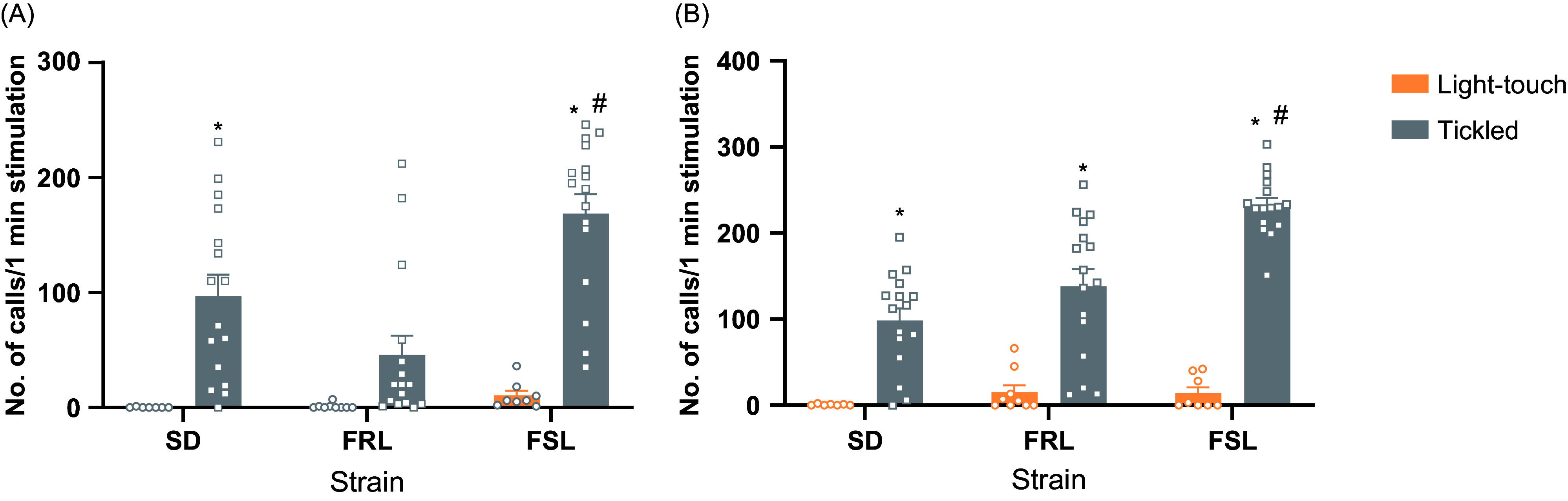
Differences in the no. of calls emitted by animals submitted to light-touch or tickle. (A) Number of calls on day 1 by strain. (B) Number of calls on day 23 by strain. Data presented as mean ± SEM. *p < 0.05 compared to light-touched of the same strain. #*p* < 0.05 compared to SD and FRL.

The number of calls was significantly different among strains and stimulation type on both days, 1 [Strain: *F* (2, 66) = 7.358, *p* = 0.0013; Stimulation: *F* (1, 66) = 47.75; *p* < 0.0001; Interaction: *F* (2, 66) = 5.334, *p* = 0.0071] and 23 [Strain: *F* (2, 66) = 11.07, *p* < 0.0001; Stimulation: *F* (1, 66) = 132.5, *p* < 0.0001; Interaction: *F* (2, 66) = 8.123, *p* = 0.0007]. The number of calls was higher in tickled animals. This difference was not statistically significant for the FRL rats on day 1 (Fig. 1). Moreover, while no differences among strains were noticed in the light-touch group, tickled FSL emitted more calls than FRL and SD on both days.

**Figure 2.**
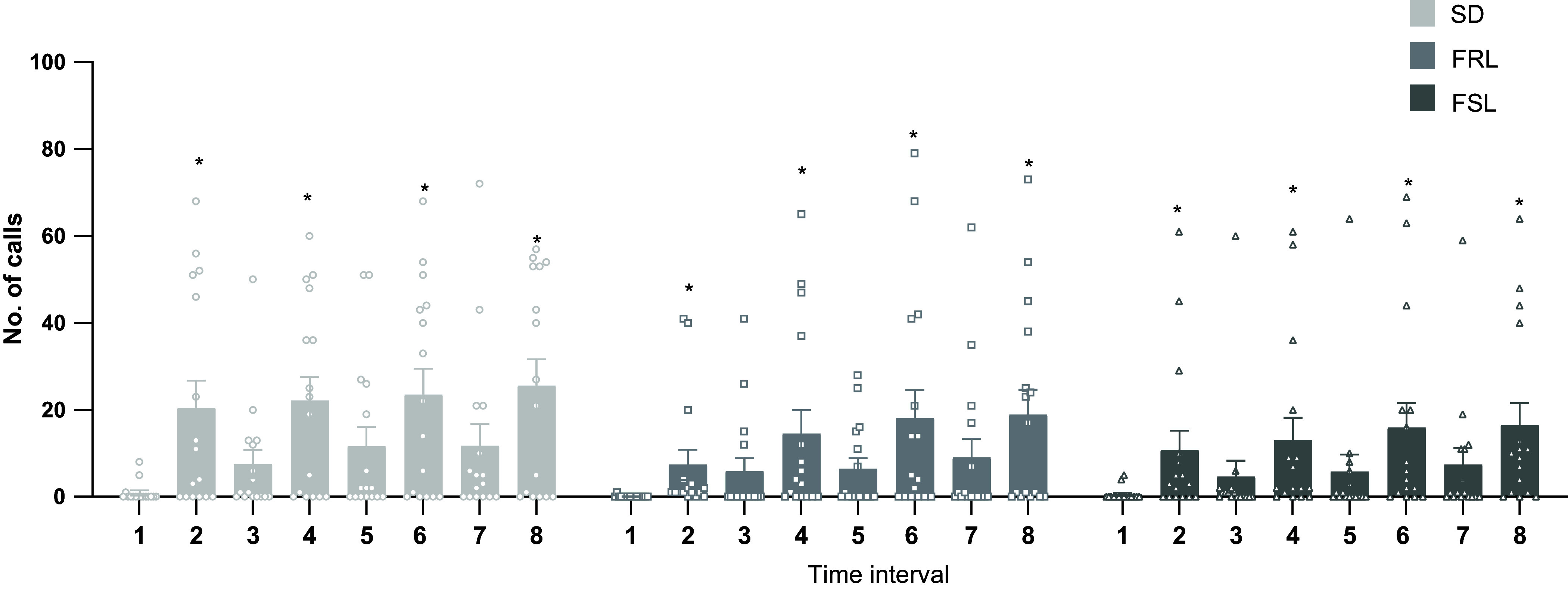
Distribution of 50 kHz calls between strains during tickling sessions on days 1 by interval of 15s. No stimulation intervals: 1, 3, 5, 7. Stimulation intervals: 2, 4, 6, 8. Data presented as mean ± SEM. *p < 0.05 compared to the previous interval.

Furthermore, in the tickled group (Fig. 2), there was a significant difference in the number of calls emitted between periods of stimulation and no-stimulation [Strain: *F* (2, 45) = 0.8199, *p* = 0.4427; Time Interval: *F* (2.707, 121.8) = 17.03, *p* < 0.0001; Interaction: *F* (14, 315) = 0.6060, *p* = 0.8599].

### The FSL rat produces more calls than their controls during tickling

**Figure 3.**
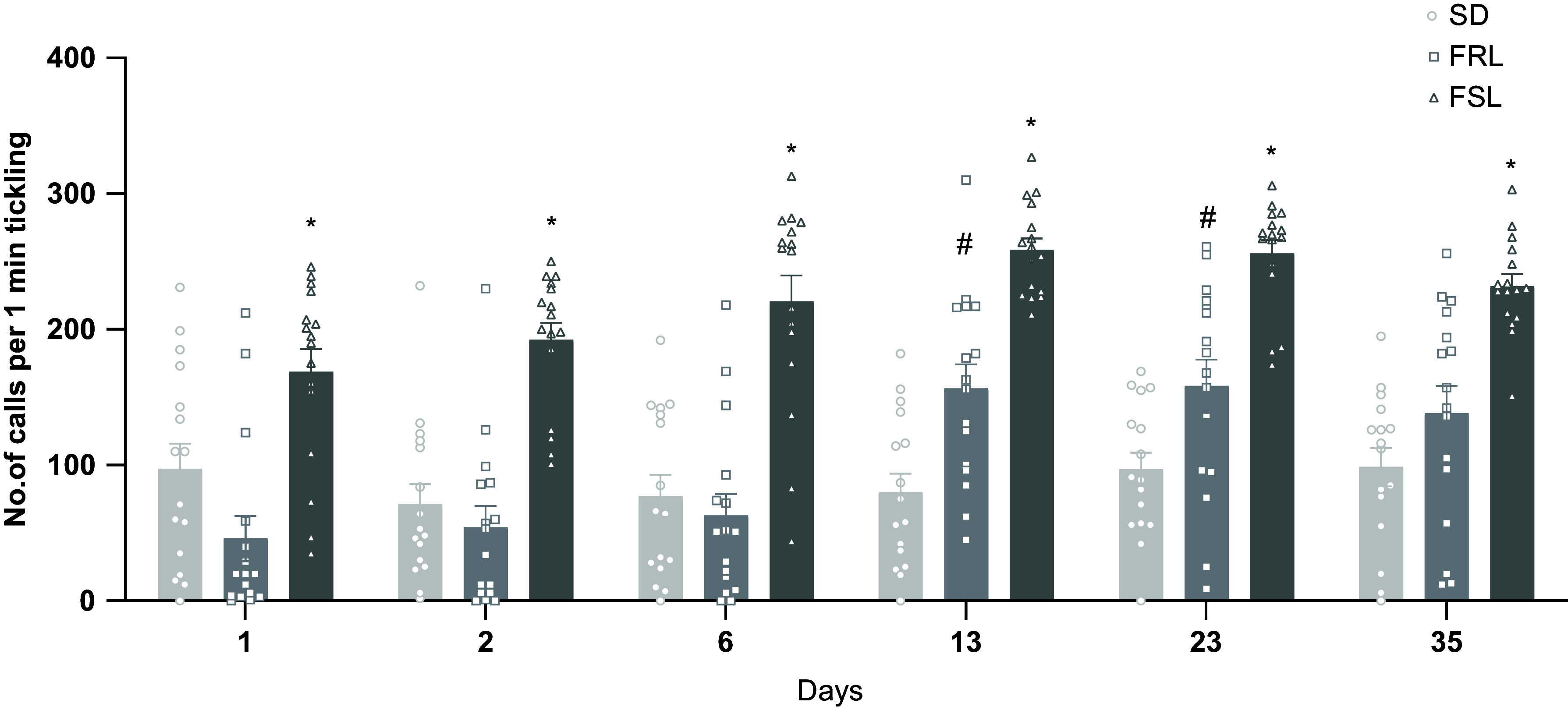
Distribution of calls across experimental days divided by strain. Data presented as mean ± SEM. *p < 0.05 FSL compared to FRL and SD, #*p* < 0.05 FRL compared to SD.

Quantitative analysis of calls during tickling revealed a significant effect of strain, day and interaction on the number of calls produced [Strain: *F* (1.991, 179.2) = 127.9, *p* < 0.0001; Day: *F* (5, 90) = 13.17, *p* < 0.0001; Interaction: *F* (10, 180) = 3.014, *p* = 0.0015]. Post hoc analysis showed that FSL emitted more calls than FRL rats. Moreover, apart from day 1, FSL also produced more calls than SD, and on days 13 and 23, FRL emitted more calls than SD (Fig. 3).

Furthermore, a correlation study was performed to evaluate whether the quantity of calls was temporally stable. FSL rats did not show a correlation between days; however, SD (from day 6) and FRL (from day 13) did (see Table 2).

**Table 2.** Stability in call profiles across days in the three strains. Correlation of calls between days across strains. FSL = Flinders Sensitive Line rat, FRL = Flinders Resistant Line rat, SD = Sprague Dawley

		Day2	Day6	Day13	Day23	Day35
FSL						
Day1	Spearmans correlation	0,658**	−0,094	0,165	0,368	0,029
	Sig. (2-tailed)	0,006	0,729	0,542	0,161	0,914
Day2	Spearmans correlation		−0,113	−0,155	0,135	0,109
	Sig. (2-tailed)		0,676	0,568	0,617	0,688
Day6	Spearmans correlation			−0,065	0,115	−0,218
	Sig. (2-tailed)			0,812	0,672	0,418
Day13	Spearmans correlation				,632**	0,483
	Sig. (2-tailed)				0,009	0,058
Day23	Spearmans correlation					0,252
	Sig. (2-tailed)					0,347
FRL						
Day1	Spearmans correlation	0,871**	0,46	−0,006	−0,102	0,037
	Sig. (2-tailed)	<0,001	0,073	0,983	0,707	0,892
Day2	Spearmans correlation		0,607*	0,258	−0,035	0,212
	Sig. (2-tailed)		0,013	0,334	0,897	0,43
Day6	Spearmans correlation			0,617*	0,331	0,244
	Sig. (2-tailed)			0,011	0,21	0,361
Day13	Spearmans correlation				0,714**	0,675**
	Sig. (2-tailed)				0,002	0,004
Day23	Spearmans correlation					0,706**
	Sig. (2-tailed)					0,002
SD						
Day1	Spearmans correlation	0,511*	0,383	0,478	0,23	0,233
	Sig. (2-tailed)	0,043	0,144	0,061	0,39	0,386
Day2	Spearmans correlation		0,524*	0,632**	0,439	0,556*
	Sig. (2-tailed)		0,037	0,009	0,089	0,025
Day6	Spearmans correlation			0,856**	0,812**	0,864**
	Sig. (2-tailed)			<0,001	<0,001	<0,001
Day13	Spearmans correlation				0,720**	0,696**
	Sig. (2-tailed)				0,002	0,003
Day23	Spearmans correlation					0,848**
	Sig. (2-tailed)					<0,001

### Qualitative call profiles among strains

**Figure 4.**
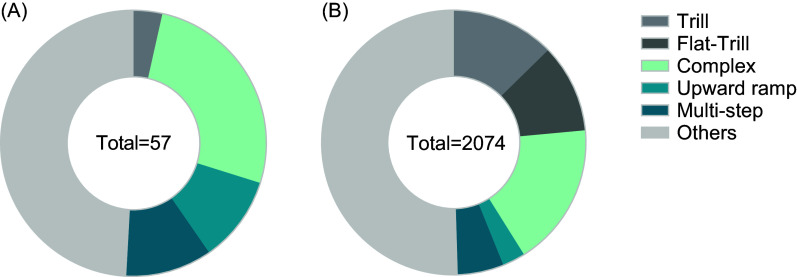
Qualitative analysis of calls in animals tickled or light-touch. (A) Distribution of calls in animals submitted to light-touch. (B) Distribution of calls in tickled animals. The three strains were merged in the two groups (tickled and light-touch).

Lastly, 57 and 2074 calls were classified in the light-touch and tickled groups, respectively. The qualitative analysis is graphically presented in Fig. 4. The percentage of calls of each type can be found in the S1 table.

**Figure 5.**
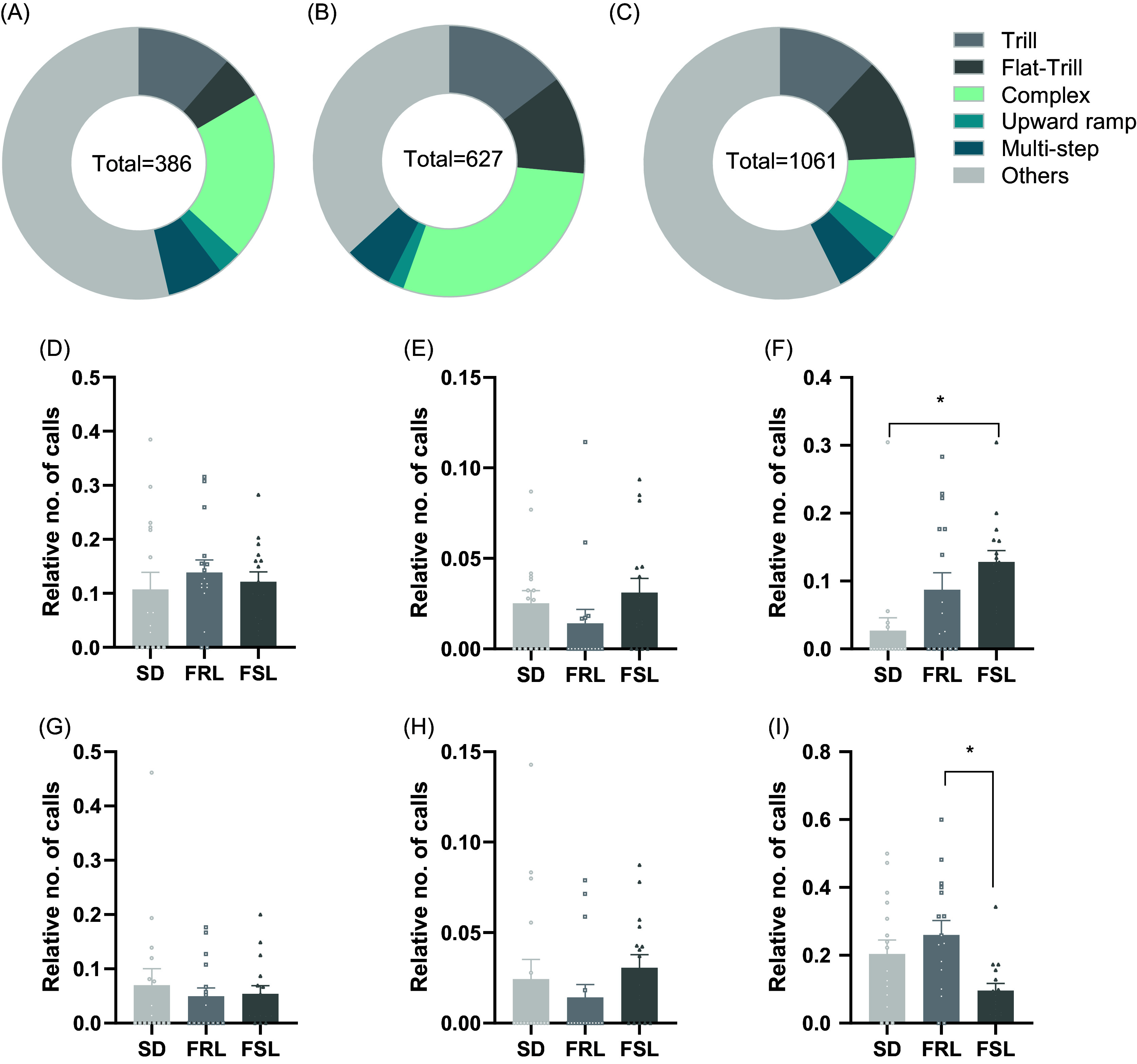
Qualitative analysis of calls in tickled animals by strain. (A) Calls distribution across categories in SD. (B) Calls distribution across categories in FRL. (C) Calls distribution across categories in FSL. (D) Relative quantity of trill calls in SD, FRL and FSL. (E) Relative quantity of flat calls in SD, FRL and FSL. (F) Relative quantity of trill-flat calls in SD, FRL and FSL. (G) Relative quantity of multistep calls in SD, FRL and FSL. (H) Relative quantity of upward ramp calls in SD, FRL and FSL. (I) Relative quantity of complex calls in SD, FRL and FSL. D-I Data presented as mean ± SEM. *p < 0.05 as indicated.

The qualitative analysis of the calls for each strain in the tickled group is graphically presented in Fig. 5A-C. The percentage of each call type can be found in Table S2. No differences in the relative number of calls were found among strains for trill [Figure 5D: *F* (2, 45) = 0.3944, *p* = 0.6764], flat [Figure 5E: *F* (2, 45) = 1.308, *p* = 0.2804], multistep [Figure 5G: *F* (2, 45) = 0.2524, *p* = 0.7780] and upward ramp calls [Figure 5H: *F* (2, 45) = 0.9351, *p* = 0.4000]. However, significant strain differences were observed in flat-trill [Figure 5F: *F* (2, 45) = 6.162, *p* = 0.0043] and complex calls [Figure 5I: *F* (2, 45) = 5.164, *p* = 0.0096].

## Discussion

This is the first time ultrasonic calls emitted by FSL during tickling have been investigated. Our results showed that I) tickling is a suitable procedure to induce 50 kHz vocalisation in rats and II) the ultrasonic response of tickled FSL rats significantly differs from FRL and SD rats.

This study demonstrated a significant difference in the number of calls produced by the tickled rats compared to the light-touch group, which is in line with previous reports (Panksepp, 1999; Panksepp & Burgdorf, 2000; Burgdorf *et al.,* 2001; Hori *et al.,* 2013). Furthermore, when evaluating the subtypes of calls emitted, the tickled groups produced trill and flat-trill combinations, whereas the light-touch groups did not produce any flat-trill combinations and only very few trill calls. These call types were previously related to tickling (Schwarting *et al*., 2007) and other rewarding stimuli like psychostimulants (Ahrens *et al*., 2009; Simola *et al*., 2010, Wright *et al*., 2010). Furthermore, previous reports have shown that pair-tested rats presented a higher proportion of trill calls compared to single-tested rats after saline or amphetamine treatment (Wright *et al*., 2010). Despite the rats in our study being single-housed, we observed increased calls after tickling.

Furthermore, tickled FSL rats produced significantly more calls than FRL and SD controls. This contrasts with the pattern observed in other animal models of depression, which showed fewer USV calls (Mallo *et al*., 2009, Rao & Sadananda, 2015, Burke *et al*., 2021). The increase in 50 kHz calls observed in juvenile FSL suggests higher sensitivity to rewarding stimulus, which contradicts the core anhedonic state found in depression. However, animal models of depression often resemble some but not all depression features (Gururajan *et al*., 2019). For instance, whether FSL can emulate anhedonia is a matter of controversy. Previous reports showed mixed findings in adult or prepubertal FSL (Pucilowski *et al*., 1993; Matthews *et al*., 1996; Malkesman *et al*., 2005; Sanchez *et al*., 2018). However, when exposed to specific environmental stressors, anhedonia was observed in FSL, perhaps revealing a heightened sensitivity to external stimuli (Pucilowski *et al*., 1993). Taken together, FSL seems more sensitive to reward and aversive interventions. In agreement with the enhanced sensitivity of FSL, we found an increase in flat-trill combinations: previous data showed that flat-trill combinations induced by reward stimulus (playing, amphetamines) were only increased in pair-tested animals and not in single animals. At the same time, FSL enhanced this call type compared to the other strains (Wright *et al*., 2010). Curiously, in humans, high environmental sensitivity during childhood may constitute a risk factor for developing depression as an adult (Lionetti *et al*., 2022). Furthermore, the vulnerability aspects of sensitivity to reward are associated with high expression of traits such as FEAR, SADNESS and ANGER (Pulver *et al*., 2020) of the Affective Neuroscience Personality Model (Davis & Panksepp, 2011). These emotional traits serve as depression vulnerability mediators in adverse environments. Consistently, it has been demonstrated in rats that higher reward sensitivity corresponds to higher sensitivity to chronic mild stress (Koiv *et al*., 2019). Single housing may additionally have been the stressor to precipitate 50 kHz USV response from the more reward-sensitive FSL.

Lastly, we performed a correlational analysis to determine whether the quantity of calls produced by a rat on one day was associated with the number of calls produced on later experimental days. Significant temporal stability was found for the SD rat from day 6 and the FRL rat from day 13. However, the number of calls emitted by FSL was not associated with the number of calls emitted on later days. A previous study on the temporal stability of the USVs produced during tickling stimulation in Wistar rats showed the calls from the beginning of the second week of stimulation to be associated with calls produced on later experimental days (Mallo *et al*., 2007). The apparent instability in the number of calls produced between days in the FSL rat may thus be a characteristic of this specific phenotype and may relate to their reward sensitivity as a representation of depression vulnerability. One could speculate that this reflects a heightened sensitivity to the tickling stimulation. Hence, minor variations in the stimulation between days may lead to more significant differences in the number of calls produced in the FSL rats compared to the SD and FRL rats.

Thus, instead of a depressive-like phenotype, the FSL rat may present a hypersensitive phenotype, which displays a heightened sensitivity to its environment regardless of whether the stimuli are appetitive or aversive. Thus, our findings have important implications for future studies of the FSL rat within the field of depression. If the depressive-like phenotype is to be modelled, it may be advantageous to expose the FSL rat to an environmental stressor. Moreover, emotional hyperreactivity was related to bipolar disorder in patients (M’bailara *et al*., 2009, Henry *et al*., Henry *et al*., 2012). Considering the neurochemical profile and the behavioural phenotype of FSL, some authors have already suggested its potential as a preclinical tool for bipolar depression (Mncube *et al*., 2021). The hypersensitive trait may endorse this hypothesis. Future research should address the performance of FSL in bipolar-like tests such as amphetamine-induced hyperlocomotion, social interaction or their response to mood stabilisers. Moreover, combining environmental stress and tickling would be interesting to explore the USV profile of stressed FSL compared to controls.

## Conclusions

The current study aimed to investigate whether USVs may be utilised as an alternative non-stressful tool to assess depressive-like behaviour in rats. The main finding of this study – a profound difference in vocalisations between an animal model of depression and controls during tickling sessions – suggests that this may be a fruitful endeavour. However, more studies on USVs in other rat models of depression are needed as the FSL rat may not be the preferred rat model to use, as it apparently shows heightened reward sensitivity and hence might not model anhedonia, at least at an early age. Instead, USVs in the FSL rat may provide a readout in a model of general vulnerability.

## Supporting information

Kai et al. supplementary material Kai et al. supplementary material

## Supplementary material

The supplementary material for this article can be found at https://doi.org/10.1017/neu.2024.61.

## References

[ref1] Abildgaard A , Kern T , Pedersen O , Hansen T , Lund S and Wegener G (2021) A diet-induced gut microbiota component and related plasma metabolites are associated with depressive-like behaviour in rats. European Neuropsychopharmacology 43, 10–21. 32933808 10.1016/j.euroneuro.2020.09.001

[ref2] Ahrens AM , Ma ST , Maier EY , Duvauchelle CL and Schallert T (2009) Repeated intravenous amphetamine exposure: rapid and persistent sensitization of 50-kHz ultrasonic trill calls in rats. Behavioural Brain Research 197(1), 205–209. 18809437 10.1016/j.bbr.2008.08.037 PMC3969445

[ref3] Ardalan M , Rafati AH , Nyengaard JR and Wegener G (2017) Rapid antidepressant effect of ketamine correlates with astroglial plasticity in the hippocampus. British Journal of Pharmacology 174(6), 483–492. 28087979 10.1111/bph.13714 PMC5323512

[ref4] Arjmand S , Pedersen MV , Silva NR , Landau AM , Joca S and Wegener G (2023) Sex and estrous cycle are not mediators of S-ketamine’s rapid-antidepressant behavioral effects in a genetic rat model of depression. International Journal of Neuropsychopharmacology 26(5), 350–358. 37067203 10.1093/ijnp/pyad016 PMC10229850

[ref5] Boulanger-Bertolus J , Rincón-Cortés M , Sullivan RM and Mouly AM (2017) Understanding pup affective state through ethologically significant ultrasonic vocalization frequency. Scientific Reports 7(1), 13483. 29044126 10.1038/s41598-017-13518-6 PMC5647438

[ref6] Brudzynski SM and Pniak A (2002) Social contacts and production of 50-kHz short ultrasonic calls in adult rats. Journal of Comparative Psychology 116(1), 73–82. 11926686 10.1037/0735-7036.116.1.73

[ref7] Burgdorf J , Knutson B , Panksepp J and Ikemoto S (2001) Nucleus accumbens amphetamine microinjections unconditionally elicit 50-kHz ultrasonic vocalizations in rats. Behavioral Neuroscienc 115(4), 940–944. 10.1037//0735-7044.115.4.940 11508733

[ref8] Burgdorf J and Panksepp J (2001) Tickling induces reward in adolescent rats. Physiology & Behavior 72(1-2), 167–173. 11239994 10.1016/s0031-9384(00)00411-x

[ref9] Burke CJ , Modlinska K , Mauro MH , Aleksandrova LR , Pellis SM , Phillips AG and Euston DR (2021) A naturalistic method to test depression: anticipation of play. Behavioural Brain Research 398, 112975 33141076 10.1016/j.bbr.2020.112975

[ref10] Cryan JF and Lucki I (2002) Assessing antidepressant activity in rodents: recent developments and future needs. Trends in Pharmacological Sciences 23(5), 238–245. 12008002 10.1016/s0165-6147(02)02017-5

[ref11] Cryan JF and Slattery DA, (2007) Animal models of mood disorders: recent developments. Current opinion in Psychiatry 20(1), 1–7. 17143074 10.1097/YCO.0b013e3280117733

[ref12] Davis KL and Panksepp J (2011) The brain’s emotional foundations of human personality and the affective neuroscience personality scales. Neuroscience & Biobehavioral Reviews 35(9), 1946–1958. 21527289 10.1016/j.neubiorev.2011.04.004

[ref13] Domingos LB , Müller HK , Da Silva NR , Filiou MD , Nielsen AL , Guimarães FS , Wegener G and Joca S (2024) Repeated cannabidiol treatment affects neuroplasticity and endocannabinoid signaling in the prefrontal cortex of the flinders sensitive line (FSL) rat model of depression. Neuropharmacology 248, 109870. 38401791 10.1016/j.neuropharm.2024.109870

[ref14] Du Jardin KG , Liebenberg N , Müller HK , Elfving B , Sanchez C and Wegener G (2016) Differential interaction with the serotonin system by S-ketamine, vortioxetine, and fluoxetine in a genetic rat model of depression. Psychopharmacology 233(14), 2813–2825. 27236785 10.1007/s00213-016-4327-5

[ref15] Dwyer JM , Duman RS (2013) Activation of Mammalian target of rapamycin and synaptogenesis: role in the actions of rapid-acting antidepressants. Biological Psychiatry, 73, 1189–1198. 10.1016/j.biopsych.2012.11.011 PMC3622786 23295207

[ref16] Gigliucci V , O’Dowd G , Casey S , Egan D , Gibney S and Harkin A (2013) Ketamine elicits sustained antidepressant-like activity via a serotonin-dependent mechanism. Psychopharmacology 228(1), 157–166. 23455595 10.1007/s00213-013-3024-x

[ref17] Gururajan A , Reif A , Cryan JF and Slattery DA (2019) The future of rodent models in depression research. Nature Reviews Neuroscience 20(11), 686–701. 31578460 10.1038/s41583-019-0221-6

[ref18] Henry C , Phillips M , Leibenluft E , M’bailara K , Houenou J and Leboyer M (2012) Emotional dysfunction as a marker of bipolar disorders. Frontiers in Bioscience E4(7), 2622–2630. 10.2741/e578 PMC3927326 22652673

[ref19] Hori M , Shimoju R , Tokunaga R , Ohkubo M , Miyabe S , Ohnishi J , Murakami K and Kurosawa M (2013) Tickling increases dopamine release in the nucleus accumbens and 50 kHz ultrasonic vocalizations in adolescent rats. Neuroreport 24(5), 241–245. 23399995 10.1097/WNR.0b013e32835edbfa

[ref20] Kaidbey JH , Ranger M , Myers MM , Anwar M , Ludwig RJ , Schulz AM , Barone JL , Kolacz J and Welch MG (2019) Early life maternal separation and maternal behaviour modulate acoustic characteristics of rat pup ultrasonic vocalizations. Scientific Reports 9(1), 19012. 31831757 10.1038/s41598-019-54800-z PMC6908621

[ref21] Kirkedal C , Elfving B , Müller HK , Moreira FA , Bindila L , Lutz B , Wegener G and Liebenberg N (2019) Hemisphere-dependent endocannabinoid system activity in prefrontal cortex and hippocampus of the flinders sensitive line rodent model of depression. Neurochemistry International 125, 7–15. 30716357 10.1016/j.neuint.2019.01.023

[ref22] Knutson B , Burgdorf J and Panksepp J (1998) Anticipation of play elicits high-frequency ultrasonic vocalizations in young rats. Journal of Comparative Psychology 112(1), 65–73. 9528115 10.1037/0735-7036.112.1.65

[ref23] Knutson B , Burgdorf J and Panksepp J (1999) High-frequency ultrasonic vocalizations index conditioned pharmacological reward in rats. Physiology & Behavior 66(4), 639–643. 10386908 10.1016/s0031-9384(98)00337-0

[ref24] Knutson B , Burgdorf J and Panksepp J (2002) Ultrasonic vocalizations as indices of affective states in rats. Psychological Bulletin 128(6), 961–977. 12405139 10.1037/0033-2909.128.6.961

[ref25] Koiv K , Vares M , Kroon C , Metelitsa M , Tiitsaar K , Laugus K , Jaako K and Harro J (2019) Effect of chronic variable stress on sensitization to amphetamine in high and low sucrose-consuming rats. Journal of Psychopharmacology 33(12), 1512–1523. 31208275 10.1177/0269881119856000

[ref26] Krystal JH , Kavalali ET and Monteggia LM (2024) Ketamine and rapid antidepressant action: new treatments and novel synaptic signaling mechanisms. Neuropsychopharmacology 49(1), 41–50. 37488280 10.1038/s41386-023-01629-w PMC10700627

[ref27] Lionetti F , Klein DN , Pastore M , Aron EN , Aron A and Pluess M (2022) The role of environmental sensitivity in the development of rumination and depressive symptoms in childhood: a longitudinal study. European Child & Adolescent Psychiatry 31(11), 1815–1825. 34170421 10.1007/s00787-021-01830-6 PMC9666332

[ref28] M’bailara K , Demotes-Mainard J , Swendsen J , Mathieu F , Leboyer M and Henry C (2009) Emotional hyper-reactivity in normothymic bipolar patients. Bipolar Disorders 11(1), 63–69. 19133967 10.1111/j.1399-5618.2008.00656.x

[ref29] Malkesman O , Braw Y , Zagoory-Sharon O , Golan O , Lavi-Avnon Y , Schroeder M , Overstreet DH , Yadid G and Weller A (2005) Reward and anxiety in genetic animal models of childhood depression. Behavioural Brain Research 164(1), 1–10. 16055204 10.1016/j.bbr.2005.04.023

[ref30] Mallo T , Matrov D , Herm L , Koiv K , Eller M , Rinken A and Harro J (2007) Tickling-induced 50-kHz ultrasonic vocalization is individually stable and predicts behaviour in tests of anxiety and depression in rats. Behavioural Brain Research 184(1), 57–71. 17675169 10.1016/j.bbr.2007.06.015

[ref31] Mallo T , Matrov D , Koiv K and Harro J (2009) Effect of chronic stress on behavior and cerebral oxidative metabolism in rats with high or low positive affect. Neuroscience 164(3), 963–974. 19706319 10.1016/j.neuroscience.2009.08.041

[ref32] Marchetti L , Lauria M , Caberlotto L , Musazzi L , Popoli M , Mathé AA , Domenici E and Carboni L (2020) Gene expression signature of antidepressant treatment response/non-response in flinders sensitive line rats subjected to maternal separation. European neuropsychopharmacology : the journal of the European College of Neuropsychopharmacology 31, 69–85. 31813757 10.1016/j.euroneuro.2019.11.004

[ref33] Matthews K , Baldo BA , Markou A , Lown O , Overstreet DH and Koob GF (1996) Rewarding electrical brain stimulation: similar thresholds for flinders sensitive line hypercholinergic and flinders resistant line hypocholinergic rats. Physiology & Behavior 59(6), 1155–1162. 8737906 10.1016/0031-9384(95)02212-0

[ref34] Mncube K , Möller M and Harvey BH (2021) Post-weaning social isolated flinders sensitive line rats display bio-behavioural manifestations resistant to fluoxetine: a model of treatment-resistant depression. Frontiers in Psychiatry 12, 688150. 34867504 10.3389/fpsyt.2021.688150 PMC8635751

[ref35] Neumann ID , Wegener G , Homberg JR , Cohen H , Slattery DA , Zohar J , Olovoer JDA and Mathé AA (2011) Animal models of depression and anxiety: what do they tell us about human condition. Progress in Neuro-Psychopharmacology & Biological Psychiatry 35, 1357–1375. 10.1016/j.pnpbp.2010.11.028 21129431

[ref36] Overstreet DH (1992) The flinders sensitive line rats: a genetic animal model of depression. Neuroscience and Biobehavioral Reviews 17(1), 51–68. 10.1016/s0149-7634(05)80230-1 8455816

[ref37] Overstreet DH and Wegener G (2013) The flinders sensitive line rat model of depression-25 years and still producing. Pharmacological Reviews 65(1), 143–155. 23319547 10.1124/pr.111.005397

[ref39] Panksepp J (1981) The ontogeny of play in rats. Developmental Psychobiology 14(4), 327–332. 7250521 10.1002/dev.420140405

[ref40] Panksepp JBJ (1999) *Laughing Rats? Playfull Tickling Arouses High Frequency Ultrasonic Chirpingin Young Rodents* . Cambridge: MIT Press.

[ref41] Panksepp J and Burgdorf J (2000) 50-kHz chirping (laughter?) in response to conditioned and unconditioned tickle-induced reward in rats: effects of social housing and genetic variables. Behavioural Brain Research 115(1), 25–38. 10996405 10.1016/s0166-4328(00)00238-2

[ref42] Panksepp J , Siviy S and Normansell L (1984) The psychobiology of play: theoretical and methodological perspectives. Neuroscience & Biobehavioral Reviews 8(4), 465–492. 6392950 10.1016/0149-7634(84)90005-8

[ref43] Porsolt RD , Anton G , Blavet N and Jalfre M (1978) Behavioral despair in rats - new model sensitive to antidepressant treatments. European Journal of Pharmacology 47(4), 379–391. 204499 10.1016/0014-2999(78)90118-8

[ref44] Porsolt RD , Bertin A and Jalfre M (1977a) Behavioral despair in mice: a primary screening test for antidepressants. Archives internationales de pharmacodynamie et de therapie 229(2), 327–336. 596982

[ref45] Porsolt RD , Le Pichon M and Jalfre M (1977b) Depression: a new animal model sensitive to antidepressant treatments. Nature 266(5604), 730–732. 559941 10.1038/266730a0

[ref46] Pucilowski Overstreet DH , Rezvani AH and Janowsky DS (1993) Chronic mild stress-induced anhedonia: greater effect in a genetic rat model of depression. Physiology & Behavior 54(6), 1215–1220. 8295967 10.1016/0031-9384(93)90351-f

[ref47] Pulver A , Kiive E and Harro J (2020) Reward sensitivity, affective neuroscience personality, symptoms of attention-deficit/hyperactivity disorder, and TPH2-703G/T (rs4570625) genotype. Acta Neuropsychiatrica 32(5), 247–256. 32338242 10.1017/neu.2020.18

[ref48] Rao RM and Sadananda M (2015) Strain-and context-based 50 kHz ultrasonic vocalizations and anxiety behaviour in the Wistar-Kyoto rat. Journal of Biosciences 40(3), 561–570. 26333402 10.1007/s12038-015-9534-4

[ref49] Reno JM , Marker B , Cormack LK , Schallert T and Duvauchelle CL (2013) Automating ultrasonic vocalization analyses: the WAAVES program. Journal of Neuroscience Methods 219(1), 155–161. 23832016 10.1016/j.jneumeth.2013.06.006 PMC3931607

[ref50] Sanchez C , El Khoury A , Hassan M , Wegener G and Mathé AA (2018) Sex-dependent behavior, neuropeptide profile and antidepressant response in rat model of depression. Behavioural Brain Research 351, 93–103. 29857028 10.1016/j.bbr.2018.05.029

[ref51] Schwarting RKW , Jegan N and Wöhr M (2007) Situational factors, conditions and individual variables which can determine ultrasonic vocalizations in male adult Wistar rats. Behavioural Brain Research 182(2), 208–222. 17367876 10.1016/j.bbr.2007.01.029

[ref52] Simola N , Ma ST and Schallert T (2010) Influence of acute caffeine on 50-kHz ultrasonic vocalizations in male adult rats and relevance to caffeine-mediated psychopharmacological effects. The International Journal of Neuropsychopharmacology 13(01), 123–132. 19545474 10.1017/S1461145709990113

[ref53] Slattery DA and Cryan JF (2012) Using the rat forced swim test to assess antidepressant-like activity in rodents. Nature Protocols 7(6), 1009–1014. 22555240 10.1038/nprot.2012.044

[ref54] Strenn N , Suchankova P , Nilsson S , Fischer C , Wegener G , Mathé AA and Ekman A (2015) Expression of inflammatory markers in a genetic rodent model of depression. Behavioural Brain Research 281, 348–357. 25277840 10.1016/j.bbr.2014.09.025

[ref55] Tillmann S and Wegener G (2019) Probiotics reduce risk-taking behavior in the elevated plus maze in the flinders sensitive line rat model of depression. Behavioural Brain Research 359, 755–762. 30170032 10.1016/j.bbr.2018.08.025

[ref56] Treccani G , Ardalan M , Chen F , Musazzi L , Popoli M , Wegener G , Nyengaard JR and Müller HK (2019) S-ketamine reverses hippocampal dendritic spine deficits in flinders sensitive line rats within 1 h of administration. Molecular Neurobiology 56(11), 7368–7379. 10.1007/s12035-019-1613-3 31037646

[ref57] Wegener G , Mathé AA , Neumann ID , (2012) Selectively bred rodents as models of depression and anxiety. Current Topics in Behavioral neuroscience 12, 139–187. 10.1007/7854_2011_192 22351423

[ref58] Whiteford HA , Degenhardt L , Rehm J , Baxter AJ , Ferrari AJ , Erskine HE , Charlson FJ , Norman RE , Flaxman AD , Johns N , Burstein R , Murray CJ and Vos T (2013) Global burden of disease attributable to mental and substance use disorders: findings from the global burden of disease study 2010. Lancet 382(9904), 1575–1586. 10.1016/S0140-6736(13)61611-6 23993280

[ref59] Wright Gourdon JC and Clarke PB (2010) Identification of multiple call categories within the rich repertoire of adult rat 50-kHz ultrasonic vocalizations: effects of amphetamine and social context. Psychopharmacology (Berl) 211(1), 1–13. 20443111 10.1007/s00213-010-1859-y

[ref60] Yamamuro T , Hori M , Nakagawa Y , Hayashi T , Sakamoto S , Ohnishi J , Takeuchi S , Mihara Y , Shiga T , Murakami K and Urayama O (2013) Tickling stimulation causes the up-regulation of the kallikrein family in the submandibular gland of the rat. Behavioural Brain Research 236, 236–243. 22982067 10.1016/j.bbr.2012.09.001

