# Supplementary material for: Ultrasonic vocalisations in the Flinders Sensitive Line rat, a genetic animal model of depression
Source: Acta Neuropsychiatr. 2025 Jan 23;37:e3. doi: 10.1017/neu.2024.61 (PMC13130335; doi:10.1017/neu.2024.61)
Supplement: Kai et al. supplementary material [file S0924270824000619sup001.docx]

**Table S1** │Classification of calls emitted by animals submitted to light-touch or tickled.

|  | Light-touch | Tickled |
| --- | --- | --- |
| Trill | 3.51 | 12.68 |
| Flat-Trill | 0 | 10.85 |
| Complex | 26.32 | 17.55 |
| Upward ramp | 10.53 | 2.80 |
| Multi-step | 10.53 | 5.64 |
| Others | 49.13 | 50.48 |

**Table S2** │Classification of calls emitted by SD, FRL and FSL animals

|  | SD | FRL | FSL |
| --- | --- | --- | --- |
| Trill | 11.40 | 14.67 | 11.97 |
| Flat-Trill | 5.18 | 11.80 | 12.35 |
| Complex | 20.21 | 29.03 | 9.80 |
| Upward ramp | 2.85 | 1.91 | 3.30 |
| Multi-step | 6.74 | 5.74 | 5.18 |
| Others | 53.63 | 36.84 | 57.40 |
